# Knowledge-driven genomic interactions: an application in ovarian cancer

**DOI:** 10.1186/1756-0381-7-20

**Published:** 2014-09-09

**Authors:** Dokyoon Kim, Ruowang Li, Scott M Dudek, Alex T Frase, Sarah A Pendergrass, Marylyn D Ritchie

**Affiliations:** 1 Department of Biochemistry and Molecular Biology, Center for Systems Genomics, Pennsylvania State University, University Park, Pennsylvania, USA

**Keywords:** Knowledge-driven genomic interaction, Integrative analysis, Grammatical evolution neural network, Clinical outcome prediction, Ovarian cancer

## Abstract

**Background:**

Effective cancer clinical outcome prediction for understanding of the mechanism of various types of cancer has been pursued using molecular-based data such as gene expression profiles, an approach that has promise for providing better diagnostics and supporting further therapies. However, clinical outcome prediction based on gene expression profiles varies between independent data sets. Further, single-gene expression outcome prediction is limited for cancer evaluation since genes do not act in isolation, but rather interact with other genes in complex signaling or regulatory networks. In addition, since pathways are more likely to co-operate together, it would be desirable to incorporate expert knowledge to combine pathways in a useful and informative manner.

**Methods:**

Thus, we propose a novel approach for identifying knowledge-driven genomic interactions and applying it to discover models associated with cancer clinical phenotypes using grammatical evolution neural networks (GENN). In order to demonstrate the utility of the proposed approach, an ovarian cancer data from the Cancer Genome Atlas (TCGA) was used for predicting clinical stage as a pilot project.

**Results:**

We identified knowledge-driven genomic interactions associated with cancer stage from single knowledge bases such as sources of pathway-pathway interaction, but also knowledge-driven genomic interactions across different sets of knowledge bases such as pathway-protein family interactions by integrating different types of information. Notably, an integration model from different sources of biological knowledge achieved 78.82% balanced accuracy and outperformed the top models with gene expression or single knowledge-based data types alone. Furthermore, the results from the models are more interpretable because they are framed in the context of specific biological pathways or other expert knowledge.

**Conclusions:**

The success of the pilot study we have presented herein will allow us to pursue further identification of models predictive of clinical cancer survival and recurrence. Understanding the underlying tumorigenesis and progression in ovarian cancer through the global view of interactions within/between different biological knowledge sources has the potential for providing more effective screening strategies and therapeutic targets for many types of cancer.

## Background

Cancer clinical outcome prediction using gene expression profiles has been proposed by the field of translational bioinformatics for better diagnostics, prognostics, and further therapeutics [1]. Somatic mutations and regulation abnormalities in a tumor cell cause substantial gene expression changes [2]. Expression of oncogenes or tumor suppressor genes promotes the malignant phenotype of cancer cells or inhibits cell division, development, or survival of cancer cell [2]. Thus, DNA microarray technologies have been widely used to predict clinical phenotypes such as stage, grade, metastatic status, recurrence, and patient survival in several cancers [3-5]. In terms of translational bioinformatics, accurate phenotype prediction based on the molecular signature can be used clinically to choose the best of several available therapies for a cancer patient.

However, clinical phenotype prediction based on gene expression profiles can vary between independent data sets [6,7]. One possible explanation is that previous studies were focused on identifying single genes with large main effects associated with clinical phenotypes. Thus, non-linear interactions without large main effects would be missed, i.e. complex signaling regulatory networks [8]. Another reason is that the single-gene approach is limited to elucidate the clinical phenotype since genes do not act in isolation, but rather interact with other genes in complex signaling or regulatory networks. Several studies incorporating genomic knowledge such as pathways or protein-protein interaction networks based on gene expression data have been developed to increase the predictive power of gene expression data for clinical phenotype prediction [9-12]. These studies have suggested that integrating gene expression profiles with biological knowledge to construct pre-defined features results in higher performance in clinical phenotype prediction and higher stability between different studies.

In general, most pathway analysis approaches assume that each pathway is independent of other pathways [13]. However, pathways are more likely to interact together rather than acting in isolation [14,15]. For instance, the P53 pathway can control the cell cycle pathway by regulating the expression of *P21* and can be activated by several pathways such as MAPK pathway. In addition, protein family interactions are essential to the functioning of individual cells in several ways through either domain-domain interactions or inter-chain protein interactions [16,17]. Thus, it would be valuable to get insight about possible interactions using biological knowledge such as pathways or protein families and identify how these relate to cancer clinical outcomes and cancer stage.

In this study, we demonstrate a novel approach for identifying knowledge-driven genomic interactions associated with cancer stage using grammatical evolution neural networks as part of the Analysis Tool for Heritable and Environmental Network Associations (ATHENA) software [18]. A knowledge-driven genomic interaction is defined as one that uses information from biological knowledge databases coupled with patient genomic profiles for model development. Thus, rather than working with gene expression data alone, we build a knowledge-based matrix prior to model generation. This is a new matrix where genes from a gene expression matrix are binned first into gene sets based on known biological knowledge, such as binned by specific biological pathways. Then we used ATHENA to classify clinical phenotype by combining the expression of genes binned by biological knowledge to form predictive models.

In order to test the utility of the proposed approach, we used ovarian cancer data from the Cancer Genome Atlas (TCGA). Ovarian cancer has the highest mortality among gynecological malignancies, and is the 5^th^ leading cause of cancer mortality in women in the United States [19]. Patients with ovarian cancer are likely to be diagnosed at a late stage due to its asymptomatic nature of this form of cancer, resulting in poor survival statistics [20]. Thus, improving our understanding of the pathogenesis of early-stage ovarian cancer is crucial for clinical studies to identify and evaluate biomarkers associated with early-stage ovarian cancer. Through the proposed approach, we found we could identify knowledge-driven genomic interactions using the same knowledge source, such as pathway-pathway interactions, that were predictive of cancer stage. We also investigated knowledge-driven genomic interactions across different sets of knowledge sources such as pathway-protein family interactions, by integrating different types of knowledge, and found we could also identify effective predictive models. Incorporating existing biological knowledge to identify knowledge-driven genomic interactions offers models interpretable from a biological stand point, further allowing for the possibility of governing alternative therapies that may improve outcomes.

## Methods

### Data

Normalized gene expression data from Affymetrix HT Human Genome U133 Array Plate Set in ovarian cancer was retrieved from the TCGA data portal (http://tcga-data.nci.nih.gov/). All samples in ovarian cancer from TCGA met broadly accepted quality control standards, including NUSE IQR, percentage present, and GAPDH 3’/5’ ratio [21]. RMA method was used for normalizing gene expression profiles [22]. In order to directly map gene identifiers to genomic knowledge such as pathways or Gene Ontology (GO) information, gene centric expression data was downloaded from TCGA portal. To generate gene centric expression values, remapping of probes to the human genome 36.1 was performed using affymetrix.aroma and an Affymetrix CDF file, which resulted in expression values for 12,042 genes and no missing values. TCGA is a highly biased sample of patients. Thus, we used the binary classification of early stage and late stage in ovarian cancer as the phenotype or outcome (dependent variable) since stage phenotype was available for the largest number of patients in ovarian cancer from TCGA. In the classification of early stage or late stage, ‘early stage’ represents the samples from patients diagnosed either *stage I* or *stage II*, whereas ‘late stage’ indicates patients diagnosed either *stage III* or *stage IV*. A total of 39 patients were classified as early stage and 454 patients were classified as late stage.

### Biofilter

Biofilter is a software tool that provides a convenient single interface for accessing multiple publicly available human genetic data sources [23,24]. These sources include information about the genomic locations of SNPs and genes, as well as relationships among genes and proteins such as interaction pairs, pathways and ontological categories. Biofilter uses a built-in database called the Library of Knowledge Integration (LOKI), which contains multiple public data resources.

Via Biofilter, we could use relevant biological knowledge for identifying knowledge-driven genomic interaction models. We used Kyoto Encyclopedia of Genes and Genomes (KEGG) [25], Gene Ontology (GO) [26], and Protein families database (Pfam) [27] from Biofilter as biological knowledge to identify knowledge-driven interactions associated with clinical phenotype. Results using biological knowledge can be biased when using gene sets consisting of extremely small genes [28], therefore gene sets from KEGG pathway, GO, and Pfam with more than 10 genes that had measured gene expression levels were selected for the further study. The total number of selected KEGG pathways, GO terms, and Pfam protein families were 249, 1514, and 356, respectively. Biofilter is open source and available at http://ritchielab.psu.edu.

### ATHENA

We used ATHENA, a multi-functional software package, to perform the three tasks: (1) performing feature/variable selections from categorical or continuous independent variables; (2) modeling main and interaction effects that explain or predict categorical or continuous clinical outcomes; (3) interpreting the significant models for use in further translational bioinformatics [29,30]. The current version of ATHENA has two different computational evolution modeling methods, Grammatical Evolution Symbolic Regression (GESR) and Grammatical Evolution Neural Networks (GENN). For this analysis, we used GENN as the modeling component.

**Figure 1:**
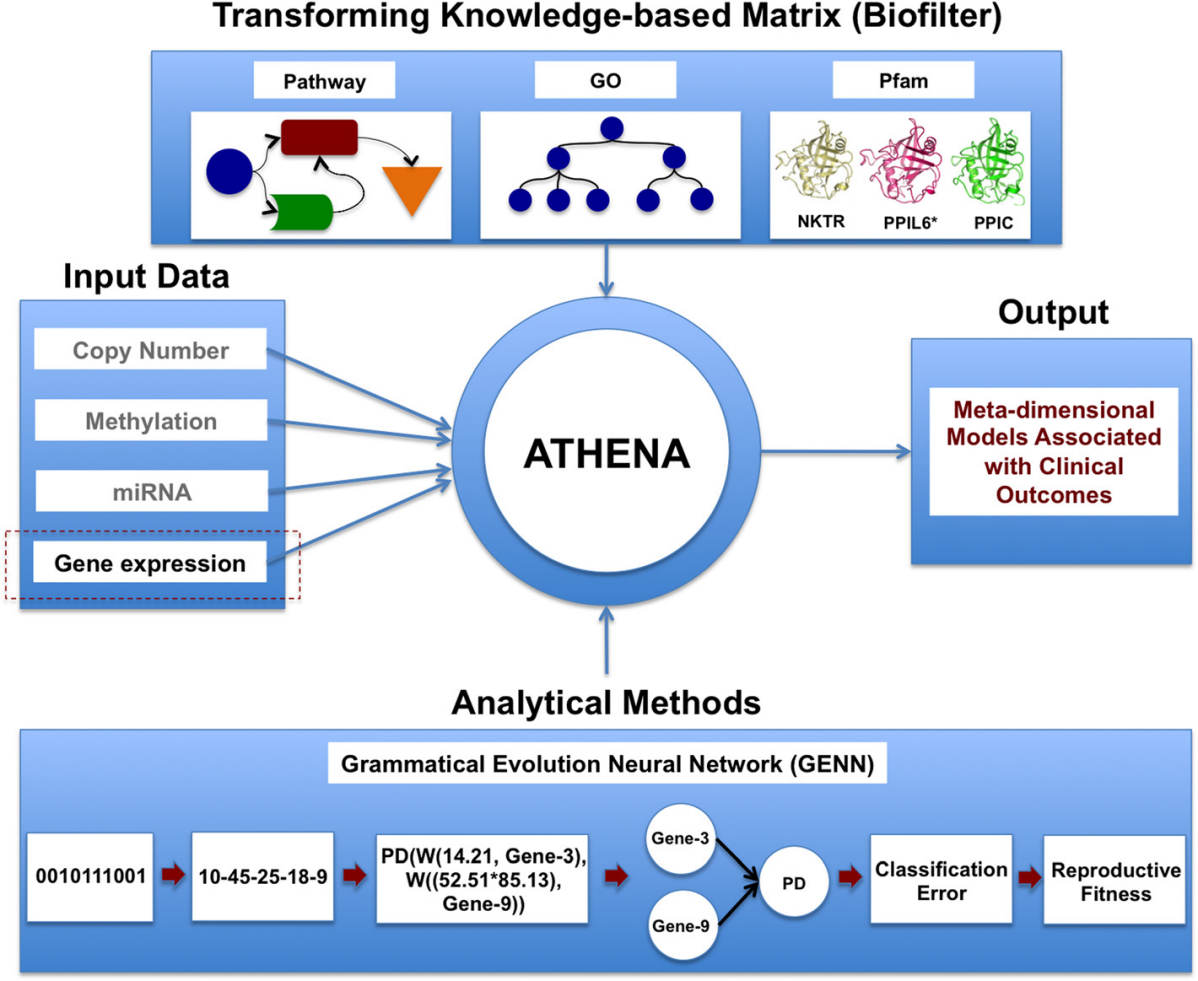
**Schematic overview of ATHENA.** ATHENA contains transformation and modeling components. The transformation component uses Biofilter, allowing researchers to transform gene-based input data into a knowledge-based matrix. Multi-omic data can be the input for developing meta-dimensional models associated with clinical outcomes of interest.

We have extended ATHENA to identify knowledge-driven genomic interactions that explain the multi-layered architecture of complex traits. Figure 1 shows a schematic of the ATHENA methodology for the current task. In particular, multi-omics data including copy number variation (CNV), methylation, miRNA, and gene expression data can be inputs for ATHENA in order to determine the meta-dimensional models of complex disease. We used gene expression data alone for the current task in order to focus on knowledge integration, instead of multi-omic data integration, for predictive model development. ATHENA is open source and available at http://ritchielab.psu.edu.

### Grammatical evolution neural networks (GENN)

In order to identify non-linear interactions between genomic features, stochastic methods employing evolutionary computing approaches utilize the full dimensionality of the data without exhaustively searching all possible combinations of variables that influence complex traits [29,31,32]. Artificial Neural Network (ANN) is a good candidate for identifying interactions that influence variance in an outcome of interest since it is able to model complex and non-linear relationships between variables. In order to optimize the input variables, weights, and network structures simultaneously, evolutionary computing approaches have been proposed [29,32]. Grammatical evolution is a flexible alternative version of genetic programming since the binary string as a heritable material can be translated into a functional solution, or computer program, via a set of grammar rules (Figure 1) [32]. The details of the grammar rules were described in a previous study [32]. The GENN algorithm is briefly described as follows:

1. The original dataset is divided into 5 equal groups for 5-fold cross-validation (4/5 for training and 1/5 for testing dataset).

2. Training begins by generating a random population of binary strings initialized to be functional ANNs. The total population is divided into demes as sub-populations across a user-defined number of CPUs for parallelization.

3. The ANNs in the population are evaluated using the training data and the fitness (balanced classification accuracy) for each model is recorded. A new population is generated as the solutions with the highest fitness are selected for crossover and reproduction.

4. Step 3 is repeated for a pre-defined number of generations. Migration of best solutions occurs between demes every n-number of generations, as specified by user.

5. The overall best solution across generations is tested using the remaining 1/5 test dataset and fitness is recorded.

6. Steps 2–5 are repeated four more times, each time using a different 4/5 of the data for training and 1/5 for testing. The best model is defined as the model identified the most over all five cross-validations.

### Developing knowledge-based datasets

**Figure 2:**
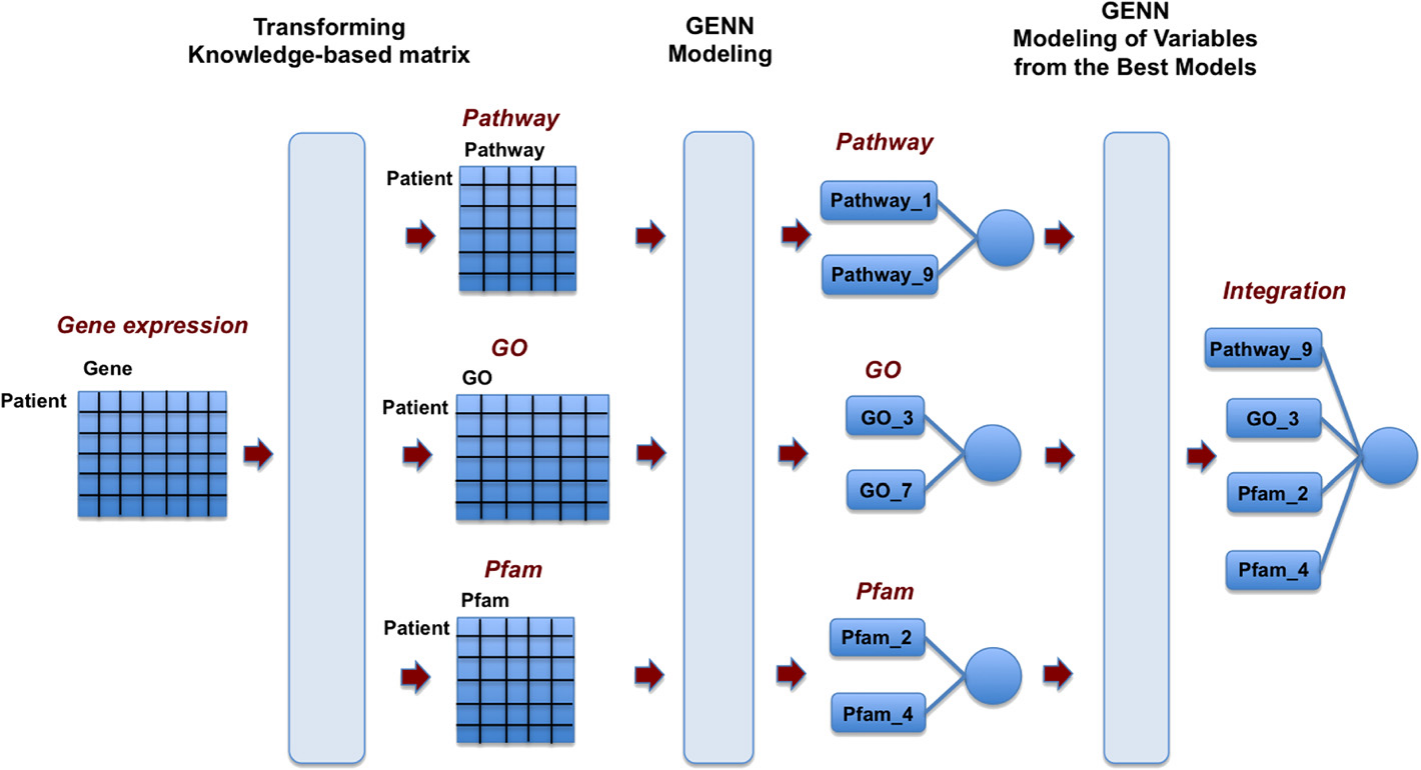
**Schematic overview of the pipeline for the analysis.** Light blue vertical bars represent each step in the pipeline: (1) Transformation of a gene expression matrix to a pathway-based, GO-based, and Pfam-based matrix (2) GENN modeling (3) GENN modeling of variables from the best models of each knowledge-based data set.

Figure 2 shows the overview of the analysis pipeline, which consists of a transformation step and a modeling step. For the transformation step, the gene expression matrix was transformed into pathway-based, GO-based, and Pfam-based matrices, that we call *knowledge*-*based datasets*. First, genes in gene expression data were mapped to pathways. Second, genes found within a concomitant pathway were grouped together and this process was repeated for all genes/pathways. Third, for each patient, each set of genes becomes a new matrix feature by summing up gene expression values across genes belonging to each pathway. Last, for each set of genes, the value of the pathway feature was divided by the total number of gene members in a pathway because the values of pathway feature can be biased toward the total number of gene members belonging to each pathway. The left side of Figure 2 shows a representation of the development of these knowledge-based matrices.

After the transformation step, we analyzed the knowledge-based datasets to generate the best predictive model using GENN. Finally, we integrated the best model from different knowledge-based datasets to develop a model associated with ovarian cancer stage to form *knowledge integration* knowledge-driven models. The balanced accuracy, which avoids inflated performance estimates on imbalanced datasets, was used in GENN as a fitness function.

## Results and discussion

### GENN modeling for identifying knowledge-driven genomic interactions

**Figure 3:**
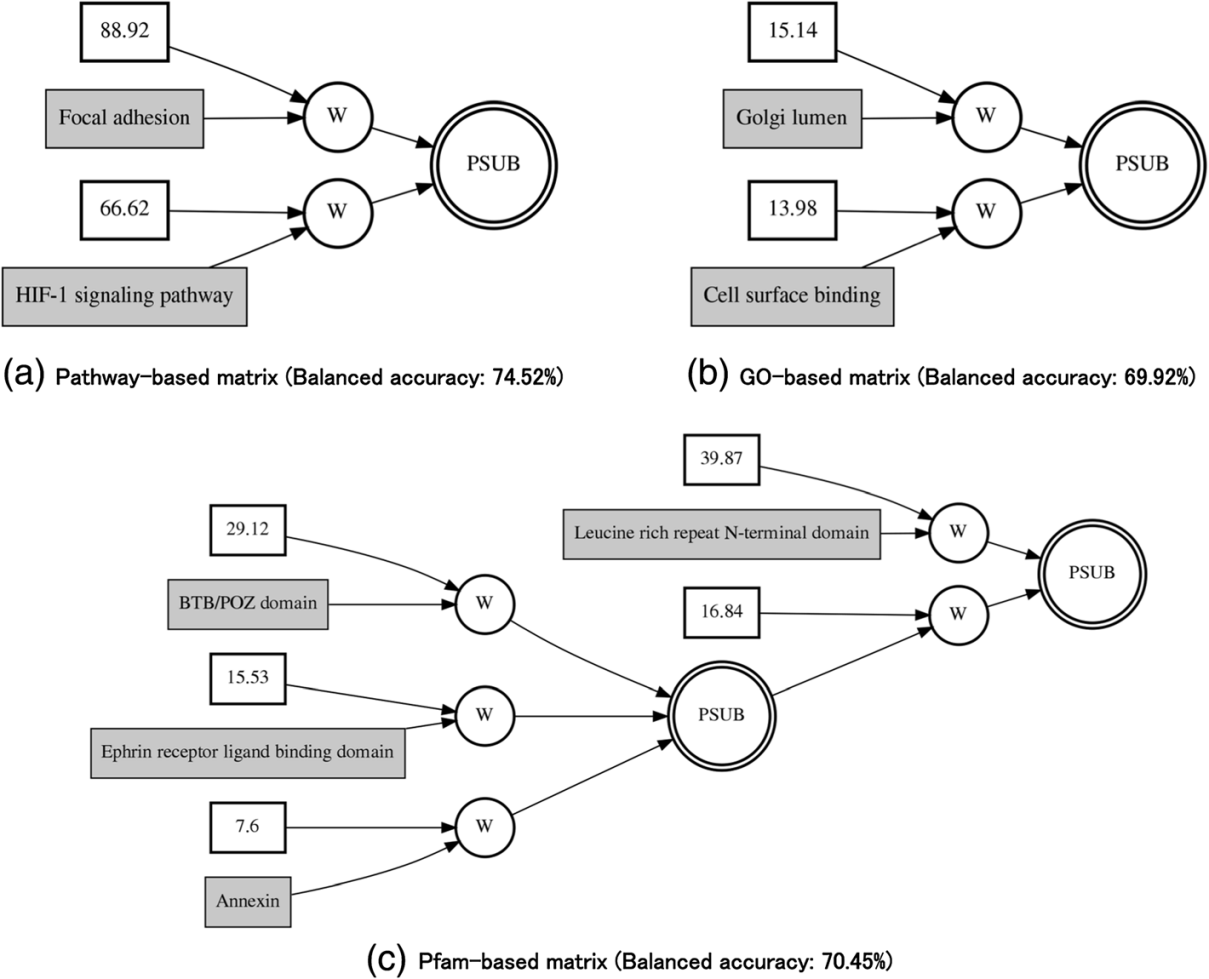
**Best GENN models from each knowledge-based dataset.** PSUB is a subtraction activation node. Constants in the white boxes are weights. Knowledge features such as pathway, GO, and Pfam, are shown in the gray boxes. **(a)** KEGG pathway-based matrix **(b)** GO-based matrix **(c)** Pfam-based matrix.

The gene expression data and knowledge-based data after the transformation step were analyzed separately by GENN with the following parameters, population sizes of 25,000 per deme, 20 demes (populations), 300 generations, and migration every 15 generations. To establish a baseline evaluation of the performance of gene expression data alone using GENN for model development, we found the best balanced accuracy value from the testing cross-validation set for each of the models with gene expression alone was 69.57%. However, when we used the knowledge-driven approach, we found an improvement in balanced accuracy. For the KEGG pathway-based matrix, GO-based matrix, and Pfam-based matrix the highest balanced accuracy increased to 74.52%, 69.92%, and 70.45%, respectively (Table 1).GENN is an evolutionary computing approach to evolve neural networks for predicting the clinical outcomes of interest by optimizing the input variables, weights, and network structure simultaneously. Thus, the final solution of GENN is the neural network. Figure 3 shows the resultant best ANN models from each knowledge-based dataset: KEGG pathway-based matrix, GO-based matrix, and Pfam-based matrix, respectively. The best models from each knowledge-based dataset showed different network structures, indicating complex interactions between knowledge features within a same biological knowledge sources.

**Table 1:** **Performance comparison between the model with gene expression data alone and models identified using knowledge**-**based matrices**

**Data type**	**Balanced accuracy**	**AUC**
Gene expression	0.6957	0.7103
Pathway	0.7451	0.7457
GO	0.6991	0.7275
Pfam	0.7046	0.7335
Integration	0.7882	0.8108

We compare here our results of evaluating gene-expression data alone, KEGG, GO, Pfam, and integration modeling. The integration model was developed by combining variables from KEGG pathway-based matrix, GO-based matrix, and Pfam-based matrix. Performances were measured based on the balanced accuracy and area under the ROC curve (AUC).

**Figure 4:**
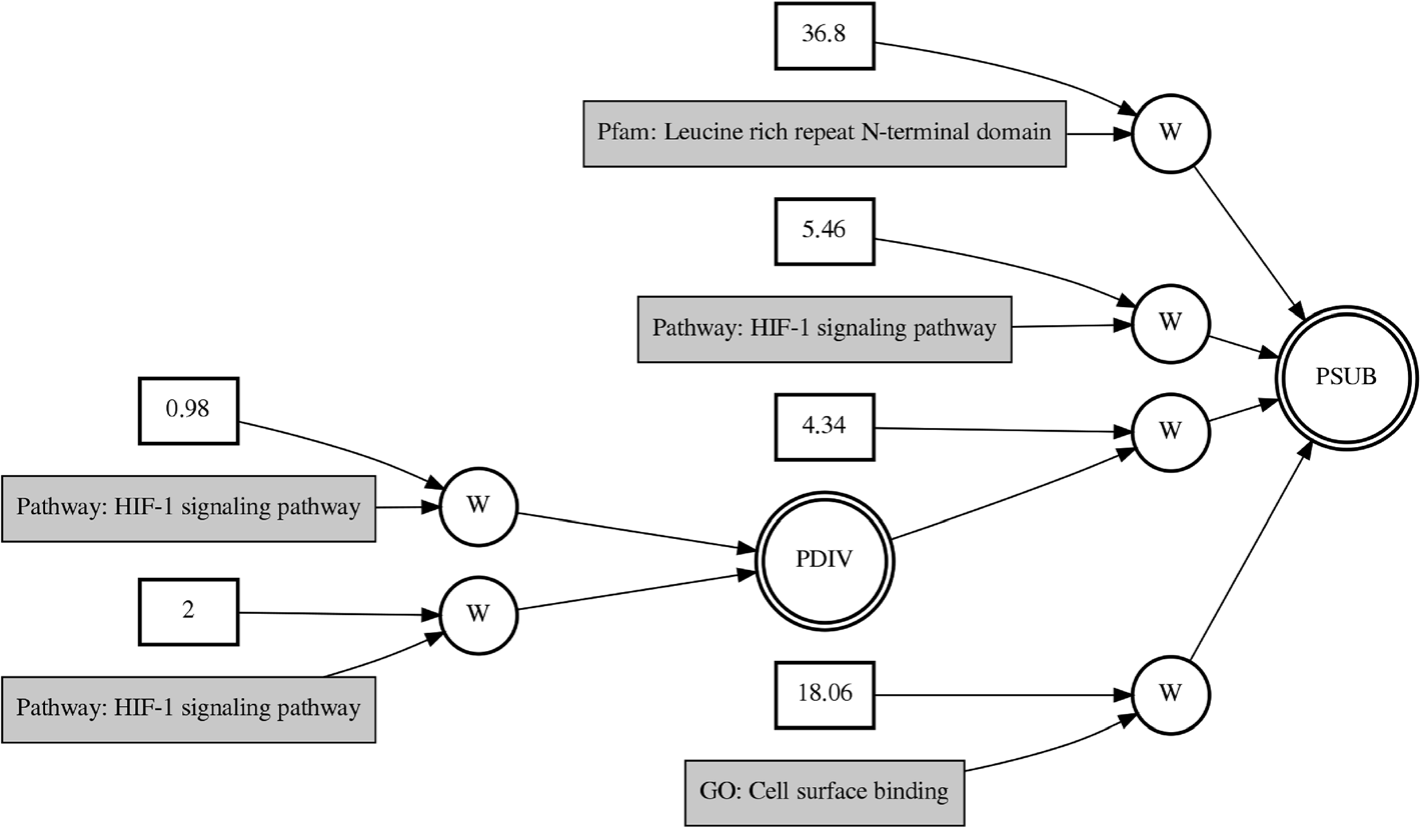
**Best GENN model from integrating knowledge-based datasets.** PSUB and PDIV represent a subtraction and division activation node, respectively. Constants in the white boxes are weights. Knowledge features such as KEGG pathway, GO, and Pfam, are shown in the gray boxes.

Further, we integrated the KEGG pathway-based matrix, GO-based matrix, and Pfam-based matrix in order to identify knowledge-driven genomic interactions between different biological knowledge sources associated with stage in ovarian cancer. The final model was created using GENN with variables from the best models of each individual knowledge-based dataset. The predictive power of integration showed the improvement compared to the models with gene expression data or single knowledge-based dataset alone (Table 1). The final model of all variables from different biological knowledge was obtained with 78.82% of balanced accuracy (Figure 4). The selected features in the final model are HIF-1 signaling pathway, cell surface binding GO term, and Pfam Leucine rich repeat N-terminal domain with variable consistency among 5 cross-validations, 4/5, 4/5, and 4/5, respectively.

### Biological interpretation

Two pathways, the “HIF-1 signaling” pathway and the KEGG “focal adhesion” pathway, were found in the GENN models associated with ovarian cancer stage. Hypoxia-inducible factor-1 (*HIF*-*1*) activates the transcription of genes that are involved in crucial aspects of cancer biology, and is well known as a cancer drug target for several cancers [33,34]. In addition, focal adhesion kinase (*FAK*), a part of the focal adhesion pathway, plays a functionally significant role in ovarian cancer migration and invasion [35]. These two cooperating pathways are thought to be important in the mechanisms of complex tumorigenesis in ovarian cancer since focal adhesion kinase signaling pathway regulates the proliferation and migration of choroidal microvascular endothelial cells by acting through *HIF*-*1* expression [36]. In addition, we found possible interactions between GO gene sets associated with ovarian cancer stage: “cell surface binding” and “golgi lumen”. Cell surface binding peptides are useful alternative agents for targeting cancer [37].

Even though models from KEGG pathway-based datasets and GO-based datasets showed linear additive effects, the models from Pfam-based dataset showed complex and non-linear interactions between protein family features associated with stage. The non-linear interactions of protein families, “annexin”, “leucine rich repeat N-terminal domain”, “BTB/POZ domain”, and “ephrin receptor ligand biding domain”, might contribute to the stage in ovarian cancer rather than any single protein family. The annexin protein family has been shown to be associated with cisplatin resistance and related to tumor recurrence in ovarian cancer [38,39]. As a member of the receptor tyrosine kinases (RTKs), elevated levels of expression and activity have been correlated with the growth of solid tumors, with Ephrin receptors of both classes A and B being over expressed in several cancers [40].

In the final model, where we used multiple knowledge sources, HIF-1 signaling from KEGG, the GO term “Cell surface binding”, and the Pfam “Leucine rich repeat N-terminal domain” were selected. Complex interactions of biological knowledge might reflect the complex molecular pathogenesis and progression of ovarian cancer. Notably, *LRRC3B*, encoding a Leucine-rich repeat-containing protein, is known as a putative tumor suppressor gene in gastric cancer and might be associated with cell surface binding peptides [41].

## Conclusions

In this study, we integrated biological knowledge to overcome the variability of diagnostic predictors across gene expression datasets and to increase the predictive power of gene expression data. Pathways co-operate together, and protein families are likely to interact with each other rather than acting in isolation, thus it is desirable to incorporate genomic knowledge for effective modeling and prediction of cancer clinical traits and outcome. Herein we proposed a novel approach for identifying knowledge-driven genomic interactions associated with cancer stage using GENN. GENN have been shown to be powerful in genetic association studies and meta-dimensional analysis of phenotypes of interest and have been shown to be successful when compared to other methods in term of prediction accuracy [29,30,32,42].

In order to demonstrate the utility of the proposed approach, ovarian cancer data from TCGA was used as a pilot project. Through the proposed approach, we identified not only knowledge interactions in a same knowledge source such as pathway-pathway interactions but also knowledge interactions among different sets of knowledge such as pathway-protein family interactions by integrating different types of knowledge. Cooperation of the “HIF-1 signaling” and “focal adhesion” pathways is thought to be important in the mechanisms of complex tumorigenesis in ovarian cancer because focal adhesion kinase signaling pathway regulates the proliferation and migration of choroidal microvascular endothelial cells by acting through *HIF*-*1* expression. In terms of accuracy, the knowledge-driven genomic interaction model outperformed the model with gene expression data alone. In addition, the results from the model were more interpretable because of the biological context of pathways. Genomic features in the same process such as signaling pathway or metabolic pathway are likely to operate together in cancer, and our modeling approach allows for models that reflect these pathways and complex interactions.

One of the limitations in this study is that the current implementation of GENN is to select the best model in the final solution because it has higher accuracy than all of the other models during the cross validation procedure. However, there might be multiple different good models and selection based on accuracy alone has its limitations. To challenge this limitation, incorporation of Pareto optimization can be alternative in the next iteration of GENN. Pareto optimization is a multi-objective optimization method that aims to maximize or minimize multiple objectives, allowing us to find multiple interactions in cancer. In addition, assessing accuracy for an independent validation set using the best model trained from the cross validations should be performed in order to estimate a true validation error. Implementing a permutation test for assessing statistical significance could be also one of the future works to overcome the current limitation from the cross validation. Furthermore, multi-omics data including SNP, CNV, methylation, miRNA, and gene expression data can be inputs for ATHENA in order to determine the meta-dimensional models of complex disease. Integrating multi-omics data and biological knowledge will be a future direction. Further, while our current study was limited to the classification of early stage or late stage in ovarian cancer, our proposed approach can be applied to other clinical outcomes such as survival, recurrence, metastasis, grade, *etc*. This methodology can be applied to other cancer types in order to identify the cancer-specific or common interactions among cancer types as well as other common, complex diseases. Understanding the underlying tumorigenesis and progression in ovarian cancer through the global view on interactions within/between different biological knowledge could provide more effective screening strategies and therapeutic targets.

## Competing interests

The authors declare that they have no competing interests.

## Authors’ contributions

DK and MDR designed and developed the study and wrote the manuscript. DK, RL, SMD, ATF, and SAP provided the experimental results and interpreted the results. MDR provided intellectual guidance and mentorship and wrote the manuscript. All authors read and approved the final manuscript.
